# Supplementary material for: Translational control by DHX36 binding to 5′UTR G-quadruplex is essential for muscle stem-cell regenerative functions
Source: Nat Commun. 2021 Aug 19;12:5043. doi: 10.1038/s41467-021-25170-w (PMC8377060; doi:10.1038/s41467-021-25170-w)
Supplement: Supplementary file 1 — Supplementary Information [file 41467_2021_25170_MOESM1_ESM.pdf]

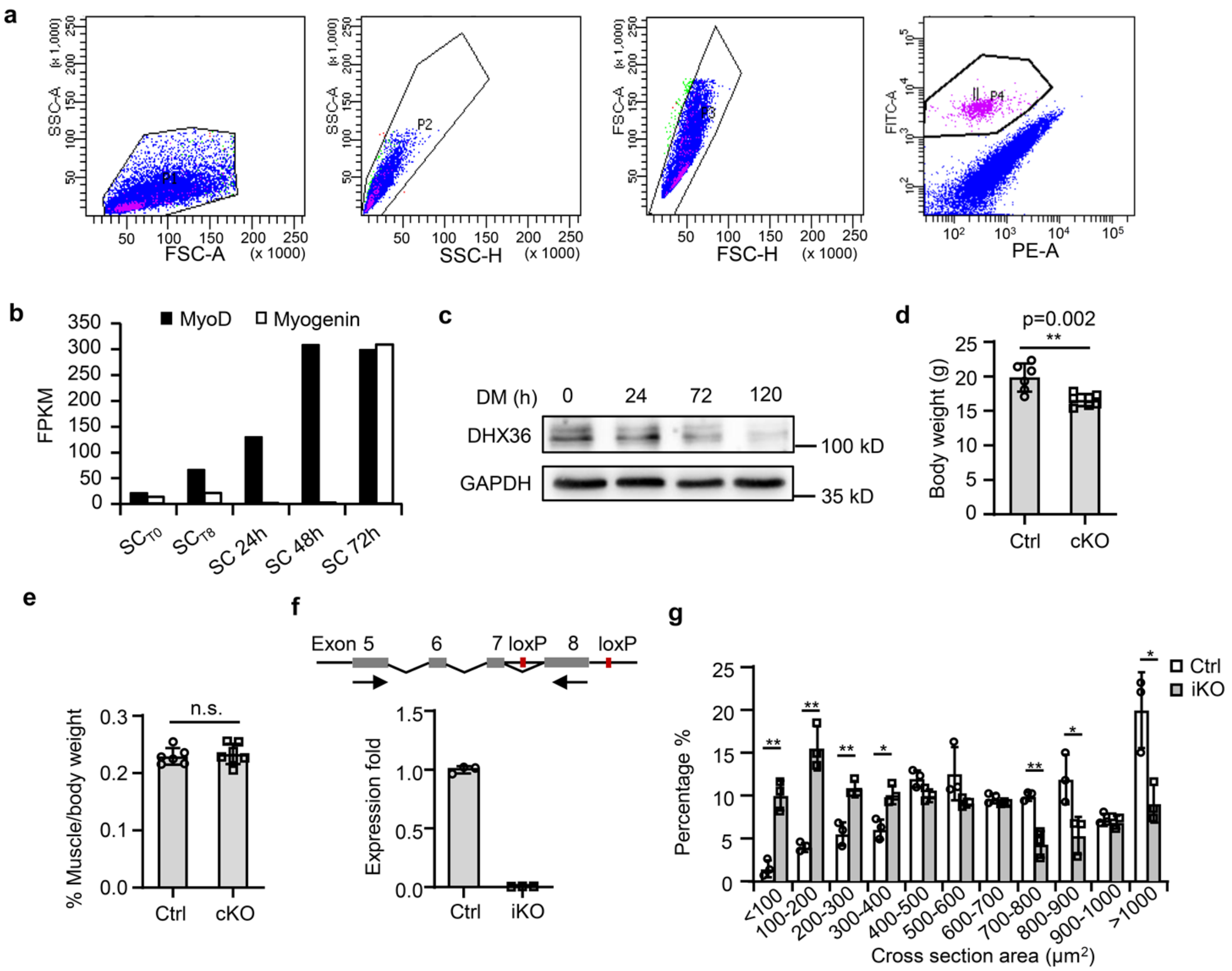
a
SSC-A
FSC-A
(x 1000)
P2
SSC-H
FSC-A
P3
FSC-H
FITC-A
P4
PE-A
b
MyoD
Myogenin
FPKM
SC T0
SC T8
SC 24h
SC 48h
SC 72h
c
DM (h)
0
24
72
120
DHX36
GAPDH
100 kD
35 kD
d
p=0.002
**
Body weight (g)
Ctrl
cKO
e
n.s.
% Muscle/body weight
Ctrl
cKO
f
Exon 5
6
7
loxP
8
loxP
Expression fold
Ctrl
iKO
g
Ctrl
iKO
Percentage %
<100
100-200
200-300
300-400
400-500
500-600
600-700
700-800
800-900
900-1000
>1000
Cross section area (μm²)

Supplementary Figure 1. DHX36 is induced in activated SCs and required for normal muscle development. a Sorting strategy for the isolation of SCs from *Pax7* nGFP mice and *Dhx36* iKO mice. P1-P4 was sequential gating and cells from p4 were collected as SCs. **b** RNA-seq derived FPKM values for *MyoD* and *Myogenin* mRNAs in SC_{T0}, SC_{T8} and SC cultured for 24h, 48h and 72h. **c** C2C12 myoblast cells were differentiated for 1, 3 and 5 days in differentiation medium (DM) and DHX36 protein level was examined by Western blot. GAPDH was used as a loading control. **d** Body weight of Ctrl and cKO female mice at similar age of 8-10 weeks, n=6 mice for Ctrl group and 7 mice for cKO group. **e** The percentage of TA muscle weight in whole body weight of Ctrl and cKO mice, n=6 mice for Ctrl group and 7 mice for cKO group. **f** Freshly isolated SCs were collected from Ctrl or iKO mice 6 days after 5 consecutive doses of Tamoxifen (Tmx) injection and cultured for 48h. *Dhx36* mRNA was examined by qRT-PCR to confirm the deletion of Dhx36. *Gapdh mRNA* was used as the normalization control. The genomic location of PCR primers used are shown on the top. n=3 independent experiments; data represent mean ± s.d.. **g** The injured TA muscles were collected from Ctrl (white bars) or iKO (grey bars) mice 7 days post injury and stained with laminin. Cross section areas (CSAs) of newly formed fibers with centrally localized nuclei were quantified, n=3 mice per group. <100 μm, P=0.0018; 100-200 μm, P=0.0015; 200-300 μm, P=0.0051; 300-400 μm, P=0.013; 700-800 μm, P=0.0043; 800-900 μm, P=0.034; >1000 μm, P=0.019. Data represent mean ± s.d. (**d, e, f, g**). Student's t-test (two-tailed unpaired) was used to calculate the statistical significance (**d**, **e**, **g**): n.s., not significant, *P < 0.05, **P < 0.01. Source data are provided as a Source Data file.

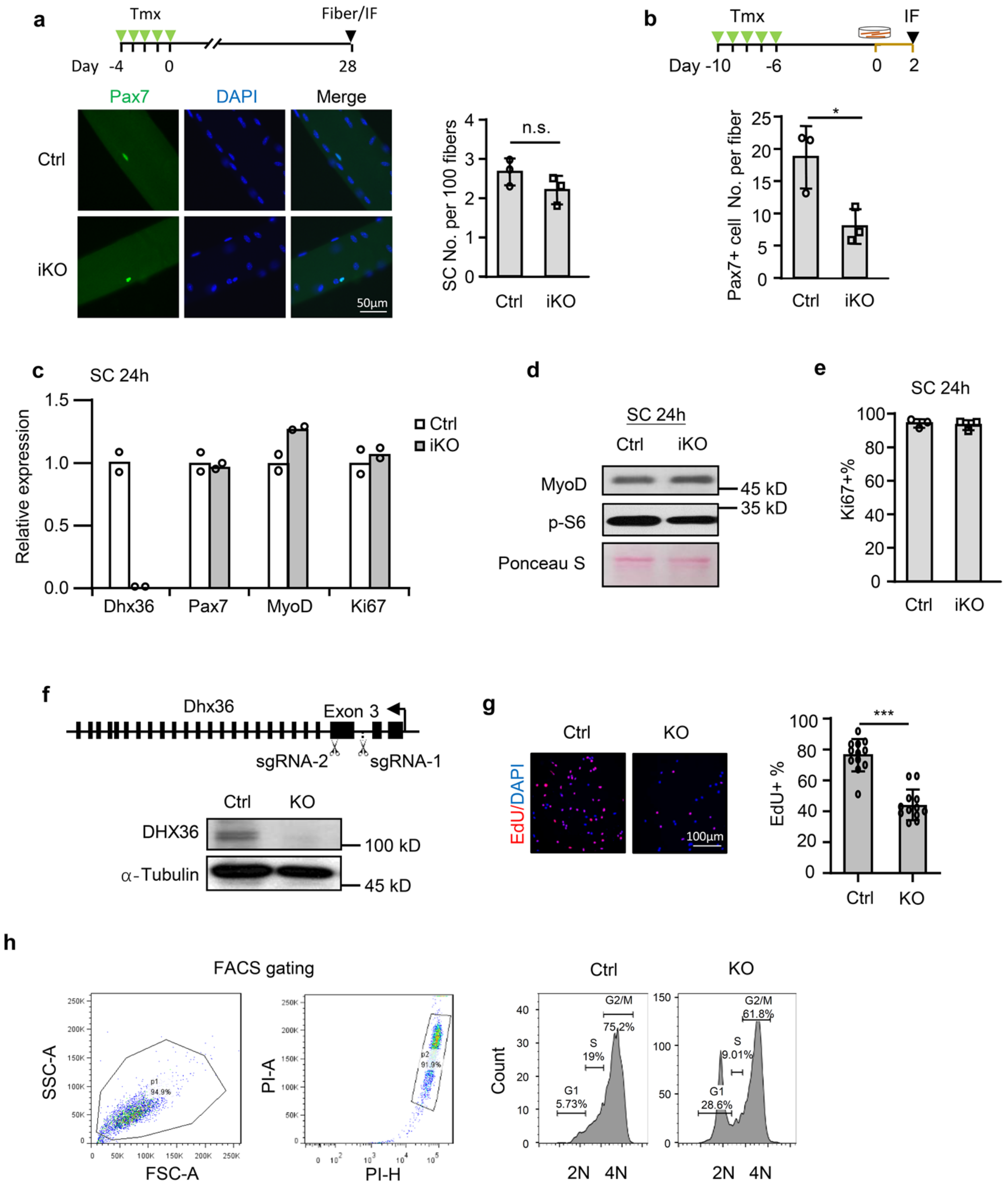
a
Tmx
Fiber/IF
Day -4 0 28
Pax7
DAPI
Merge
Ctrl
iKO
50μm
SC No. per 100 fibers
n.s.
Ctrl iKO
b
Tmx
IF
Day -10 -6 0 2
Pax7+ cell No. per fiber
*
Ctrl iKO
c
SC 24h
Relative expression
Ctrl
iKO
Dhx36 Pax7 MyoD Ki67
d
SC 24h
Ctrl iKO
MyoD
p-S6
Ponceau S
45 kD
35 kD
e
SC 24h
Ki67+%
Ctrl iKO
f
Dhx36
Exon 3
sgRNA-2
sgRNA-1
Ctrl KO
DHX36
α-Tubulin
100 kD
45 kD
g
Ctrl
KO
EdU/DAPI
100μm
EdU+ %
***
Ctrl KO
h
FACS gating
SSC-A
FSC-A
PI-A
PI-H
Ctrl
KO
Count
G1 5.73%
S 19%
G2/M 75.2%
G1 28.6%
S 9.01%
G2/M 61.8%
2N 4N
2N 4N

Supplementary Figure 2. Inducible loss of *Dhx36* in adult SCs impairs skeletal muscle regeneration. a Single myofibers were isolated from extensor digitorum longus (EDL) muscle of Ctrl or iKO mice 4 weeks after Tmx injection. IF staining of Pax7 (green) was performed on the fibers. Representative images are shown. The numbers of Pax7+ cells were counted from >15 fibers/mouse. Scale bar=50 µm; n=3 mice per group. **b** Freshly isolated myofibers were cultured for 48h and the numbers of Pax7+ cells were quantified as above; n=3 mice per group. P=0.029. **c** SCs isolated from Ctrl or iKO mice were cultured for 24h; mRNA levels of *Dhx36*, *Pax7*, *MyoD* and *Ki67* were measured by qRT-PCR. *18s* rRNA was used as the normalization control. Data represent the average of two independent experiments. **d** Protein levels of MyoD and p-S6 were examined in the above cells by Western blot. Ponceau S staining was used as the loading control. **e** Immunostaining of Ki67 was performed in the above cells and Ki67+ cells number was quantified by counting from 10 randomly chosen fields; n=3 mice per group. **f** Exon 3 of *Dhx36* was deleted via CRISPR-Cas9 in C2C12 myoblast cells. Western blot was performed to confirm the inactivation of DHX36 in KO cells with α-tubulin as the loading control. **g** EdU incorporation assay was performed in the above generated Ctrl or Dhx36 KO cells and the EdU incorporation percentage was calculated by counting from 10 randomly selected fields per cell type. Representative images are shown. Scale bar=100 µm. Data represent the average of indicated No. of independent fields ± s.d. P=0.00000012. **h** The above Ctrl or KO myoblast cells were treated with nocodazole for 3h and cell cycle analysis was performed by FACS. Gating strategy was shown in left panel. KO cells showed significant increase of 2N cell accumulation and G1 phase arrest. Data represent mean ± s.d. (**a, b, e**). Student's t-test (two-tailed unpaired) was used to calculate the statistical significance (**a, b, g**): n.s., not significant, *P < 0.05, ***P < 0.001. Source data are provided as a Source Data file.

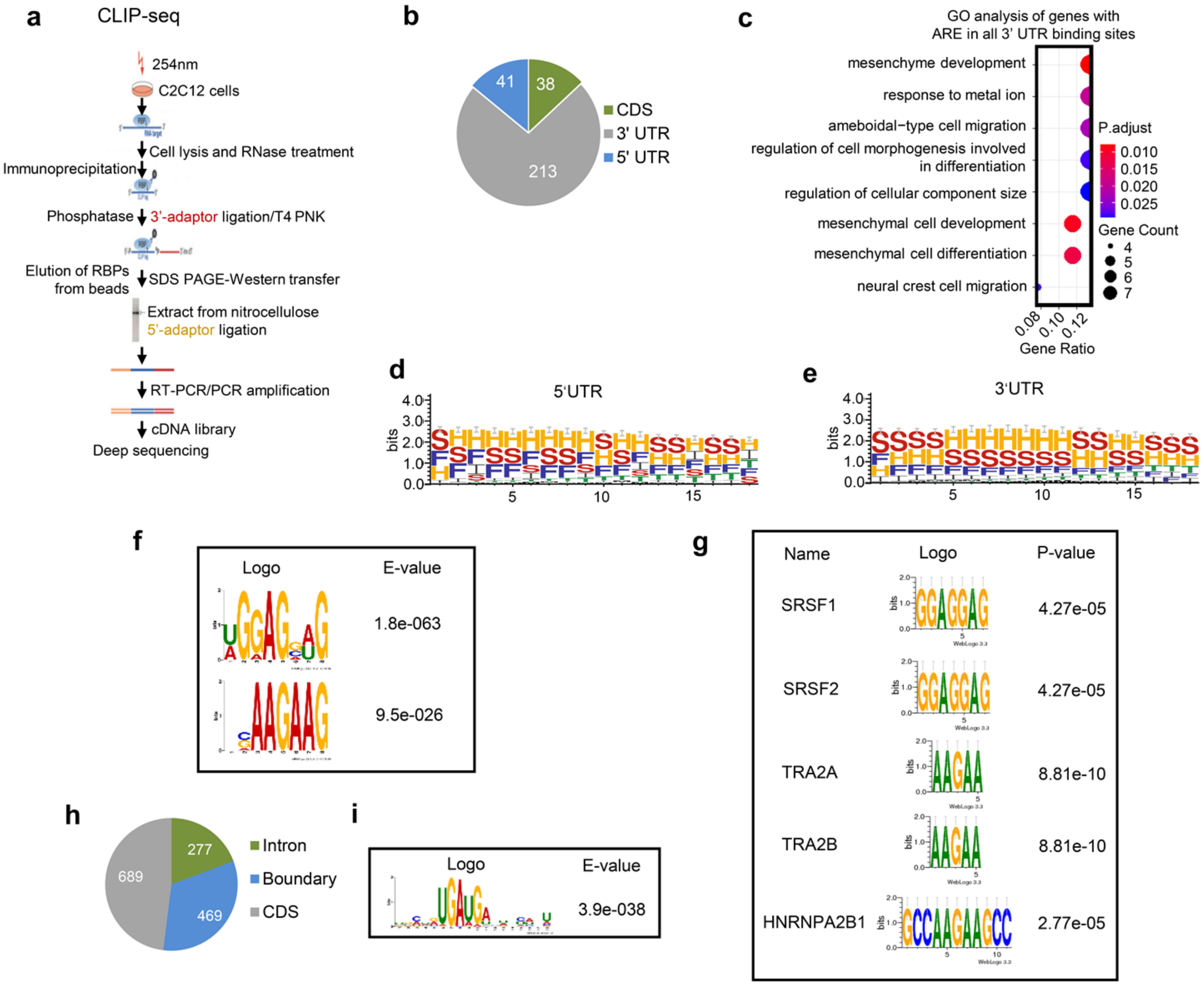

a
CLIP-seq
254nm
C2C12 cells
Cell lysis and RNase treatment
Immunoprecipitation
Phosphatase
3'-adaptor ligation/T4 PNK
Elution of RBPs from beads
SDS PAGE-Western transfer
Extract from nitrocellulose
5'-adaptor ligation
RT-PCR/PCR amplification
cDNA library
Deep sequencing
b
41
38
213
CDS
3' UTR
5' UTR
c
GO analysis of genes with ARE in all 3' UTR binding sites
mesenchyme development
response to metal ion
ameboidal−type cell migration
regulation of cell morphogenesis involved in differentiation
regulation of cellular component size
mesenchymal cell development
mesenchymal cell differentiation
neural crest cell migration
P.adjust
0.010
0.015
0.020
0.025
Gene Count
4
5
6
7
0.08
0.10
0.12
Gene Ratio
d
5'UTR
bits
e
3'UTR
bits
f
Logo
E-value
1.8e-063
9.5e-026
g
Name
Logo
P-value
SRSF1
4.27e-05
SRSF2
4.27e-05
TRA2A
8.81e-10
TRA2B
8.81e-10
HNRNPA2B1
2.77e-05
h
277
469
689
Intron
Boundary
CDS
i
Logo
E-value
3.9e-038

Supplementary Figure 3. Transcriptomic binding profiling uncovers diversified possible roles of DHX36. **a** Workflow of the DHX36 CLIP-seq experiment. **b** The number of AU-rich elements (AREs) identified in CDS (green), 5'UTR (blue) and 3'UTR (grey) regions bound by DHX36. **c** GO analysis result for genes with AREs in 3'UTR bindings ordered by gene ratio (proportion of genes annotated for each GO term). Dots are colored by adjusted P-value (degree of enrichment) and their size corresponds to the gene counts annotated to each GO term. **d-e** Structural motifs identified on motif 2 enriched sequences in DHX36 bound 5'UTR or 3'UTR regions. S: Stems, M: Multiloops, H: Hairpins, I: Internal loops, F and T: dangling start end. **f** MEME identifies the top enriched 8 nt motifs in CDS binding sites from Fig. 4j. The E-values measuring the statistical significance of each motif identified by MEME are shown. **g** The identified motifs in **f** share high similarity with the binding motifs of several RBPs associated with alternative splicing including SRSF1, SRSF2, TRA2A, TRA2B, HNRNPA2B1. **h** The number of DHX36 binding site identified on intron (green), CDS (grey) or intron-CDS boundary (blue). **i** MEME identifies a motif resembles the C box motif on C/D box snoRNAs in the above intronic binding regions not overlapping with intronic repeat elements. The E-value measuring the statistical significance of the motif identified by MEME is shown.

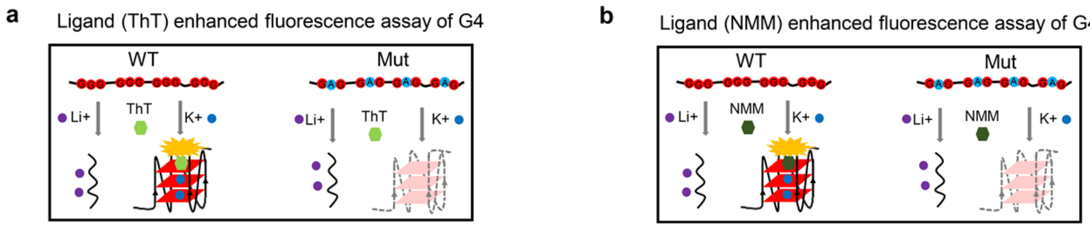

c

mRNA	Sequence (5'-3')	Predicted rG4
Mknk2	GGUGGCGGUGGUGGCGGCGGUGGCGGCGG	2-quartet
Pja2	GGCGGUGGUGGCGGGUGGAGG	2-quartet
Hnrnpl	GGCGGGGGGAGGCGGG	2-quartet
Nid2	GGAGUGGAGGGCAGAGCCAGGGAGGAG	Bulge
Cd82	GGUGAGGAGGAAGUGGGGGGUUGGG	Bulge

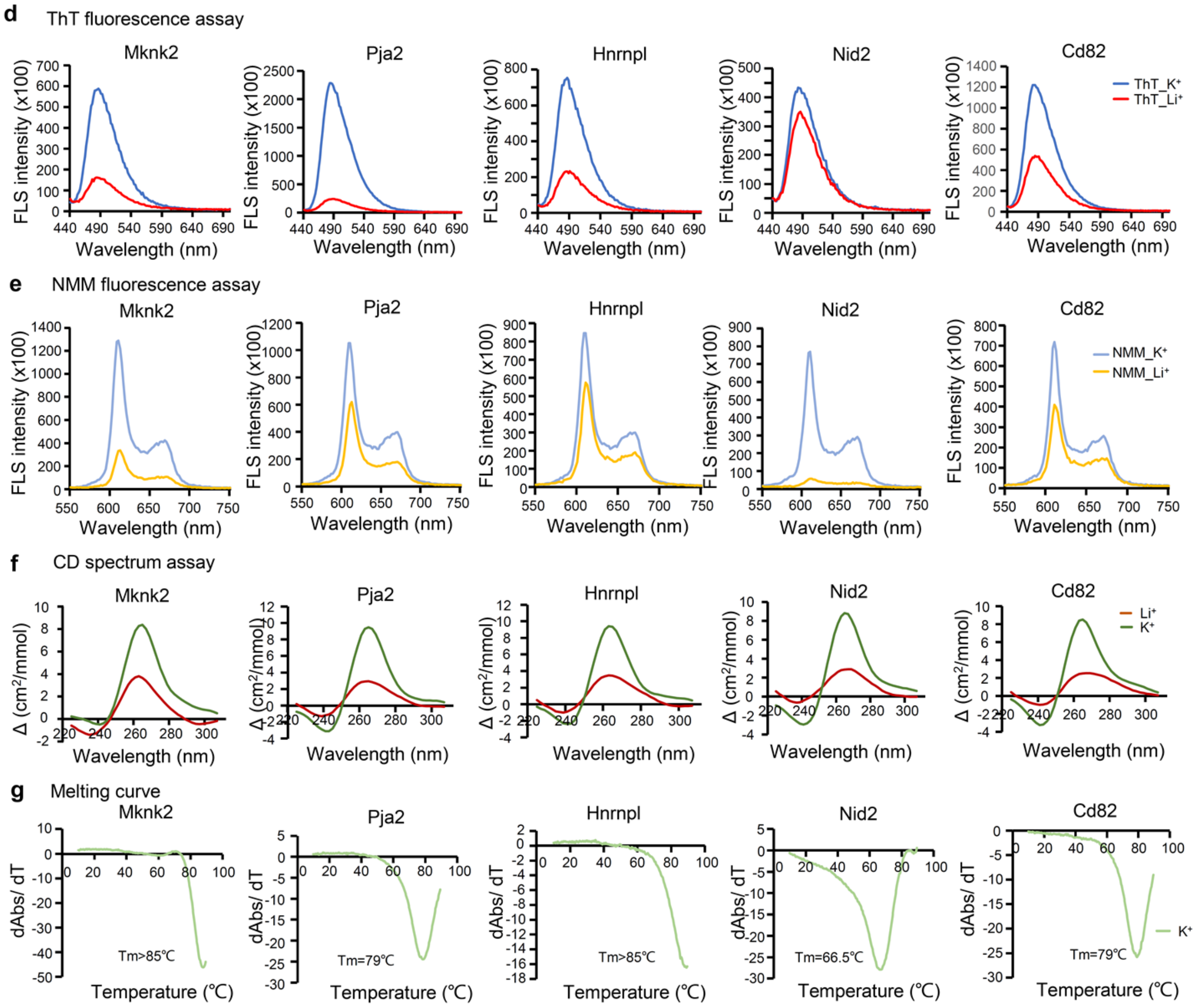

Supplementary Figure 4. Biophysical characterization of potential rG4 forming sequences. a Schematic illustration of the Thioflavin T (ThT) staining assay. **b** Schematic illustration of the N-methyl mesoporphyrin IX (NMM) staining assay. **c** Five selected mRNA sequences with predicted rG4 subtype formation. **d** RNA oligos corresponding to the predicted rG4 formation sequences in each of the above mRNAs were treated with 150 mM Li+ (red line) or K+ (blue line) together with 1 μM ThT and excited at 425 nm. Plot of the intensity of Fluorescent spectrum (FLS) with the wavelength from 440 nm to 700 nm. **e** The above oligos were stained with NMM under the treatment of 150 mM Li+ (yellow) or K+ (blue) and excited at a wavelength of 394 nm with FLS collected from 550 nm to 750 nm. **f** CD spectrum of the above oligos under 150mM KCl (green) or LiCl (red) conditions. The oligos were examined from 220 to 310 nm at a 2 nm interval, and the data were blanked and normalized to mean residue ellipticity. The data were interpreted using Spectra Manager Suite (Jasco Software). **g** UV melting assay of the above G4 forming oligos. The samples were examined at 295 nm from 5°C to 95°C with 0.5°C increment per minute. The data were blanked and smoothed over 10 data points. The melting temperature (Tm) for each oligo is indicated. Source data are provided as a Source Data file.

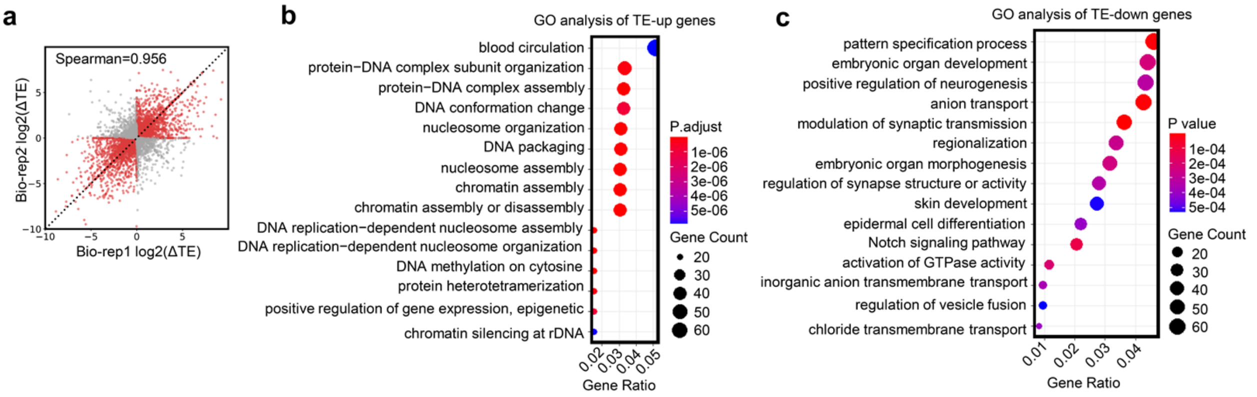

d

Gene ID	Location	ΔTE	ΔmRNA
Gnai2	chr9:107516468-107537673	0.45	1.44
Yap1	chr9:7932000-8004596	0.33	3.06
Ssbp3	chr4:106584074-106722299	0.42	1.66
Gnb1	chr4:154865469-154933378	0.49	1.52
Dazap1	chr10:79727735-79751158	0.57	1.65
Dlg1	chr16:31663528-31873442	0.59	2.17
Adam10	chr9:70526807-70628036	0.60	2.10

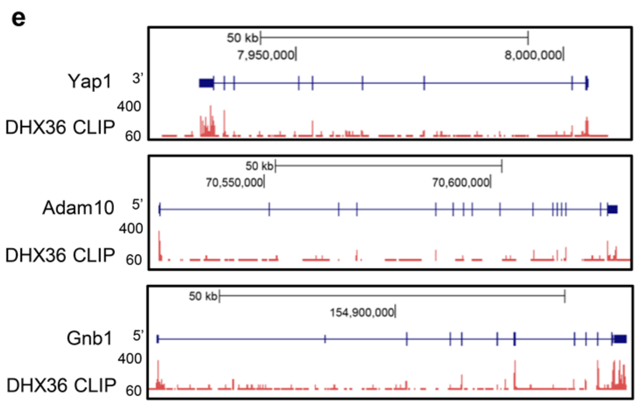

Supplementary Figure 5. Translational profiling reveals that DHX36 facilitates mRNA translation through binding to 5'UTR rG4. a The translational efficiency change in KO vs Ctrl cells (ΔTE) was calculated and the scatterplot displays the log2(ΔTE) values of mRNAs in both replicates. Spearman: Spearman Correlation Coefficient. Red dots: the genes with consistent TE change trend in two biological replicates. **b-c** GO analysis results for TE-up or TE-down genes in Fig. 5c ordered by gene ratio (proportion of genes annotated for each GO term). Dots are colored by P-value or adjusted P-value (degree of enrichment) and their size corresponds to the gene counts annotated to each GO term. **d** List of 7 targets with DHX36 binding at 5'UTR and TE down-regulation upon Dhx36 loss that were related to cell proliferation regulation. The fold change of TE and mRNA upon Dhx36 inactivation were also shown. **e** Genomic snapshot of DHX36 CLIP-seq track showing its binding at *Yap1, Adam10 and Gnb1* mRNA.

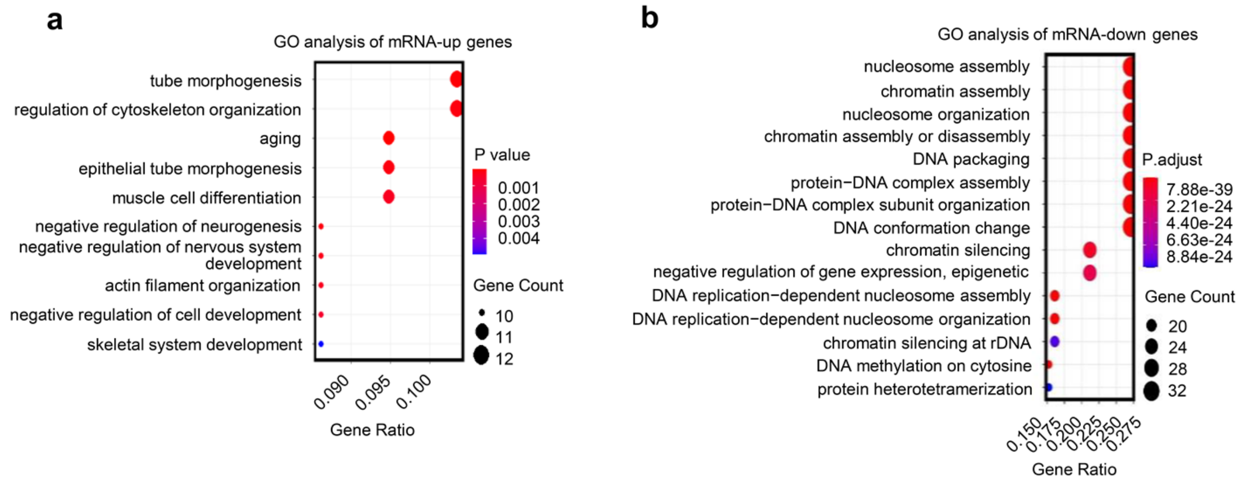

Supplementary Figure 6. DHX36 loss alters the mRNA level of genes involved in multiple biological processes. a-b GO analysis results for mRNA-up or -down genes in Figure 6a ordered by gene ratio (proportion of genes annotated for each GO term). Dots are colored by P-value or adjusted P-value (degree of enrichment) and their size corresponds to the gene counts annotated to each GO term.

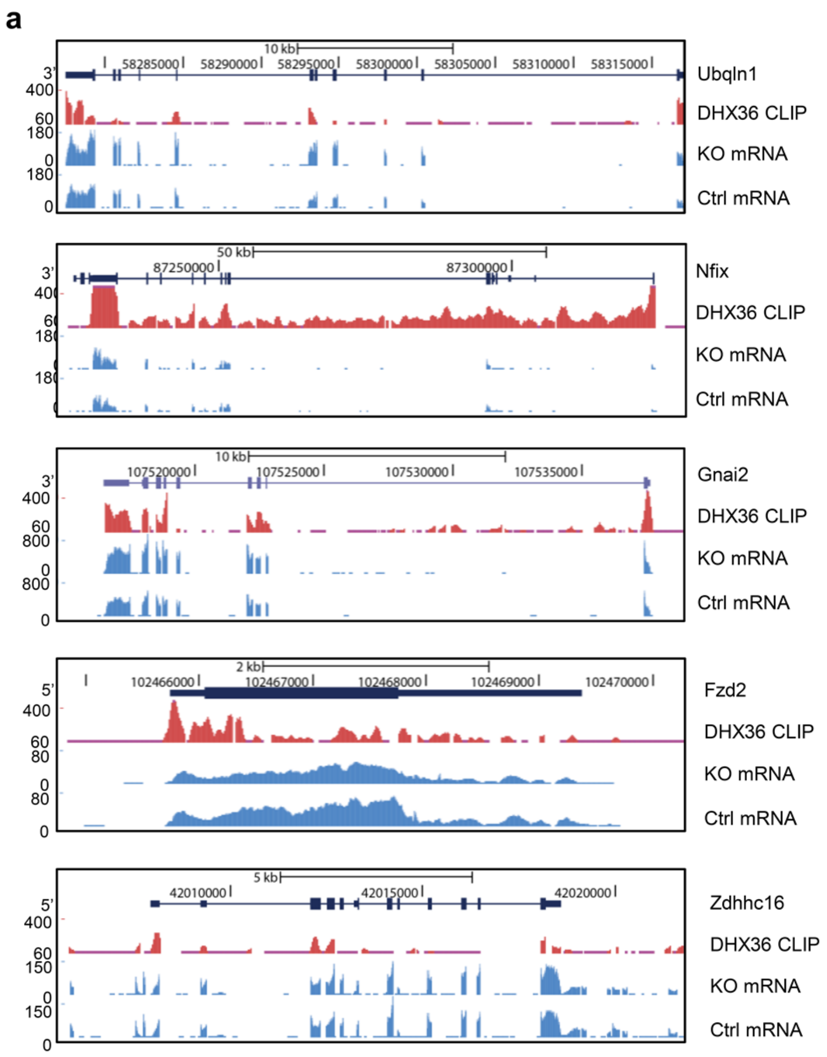

Supplementary Figure 7. Genome snapshots illustrating DHX36 binding and total mRNA abundance of five genes including *Ubqln1*, *Nfix*, *Gnai2*, *Fzd2* and *Zdhhc16* in KO vs Ctrl myoblast cells.

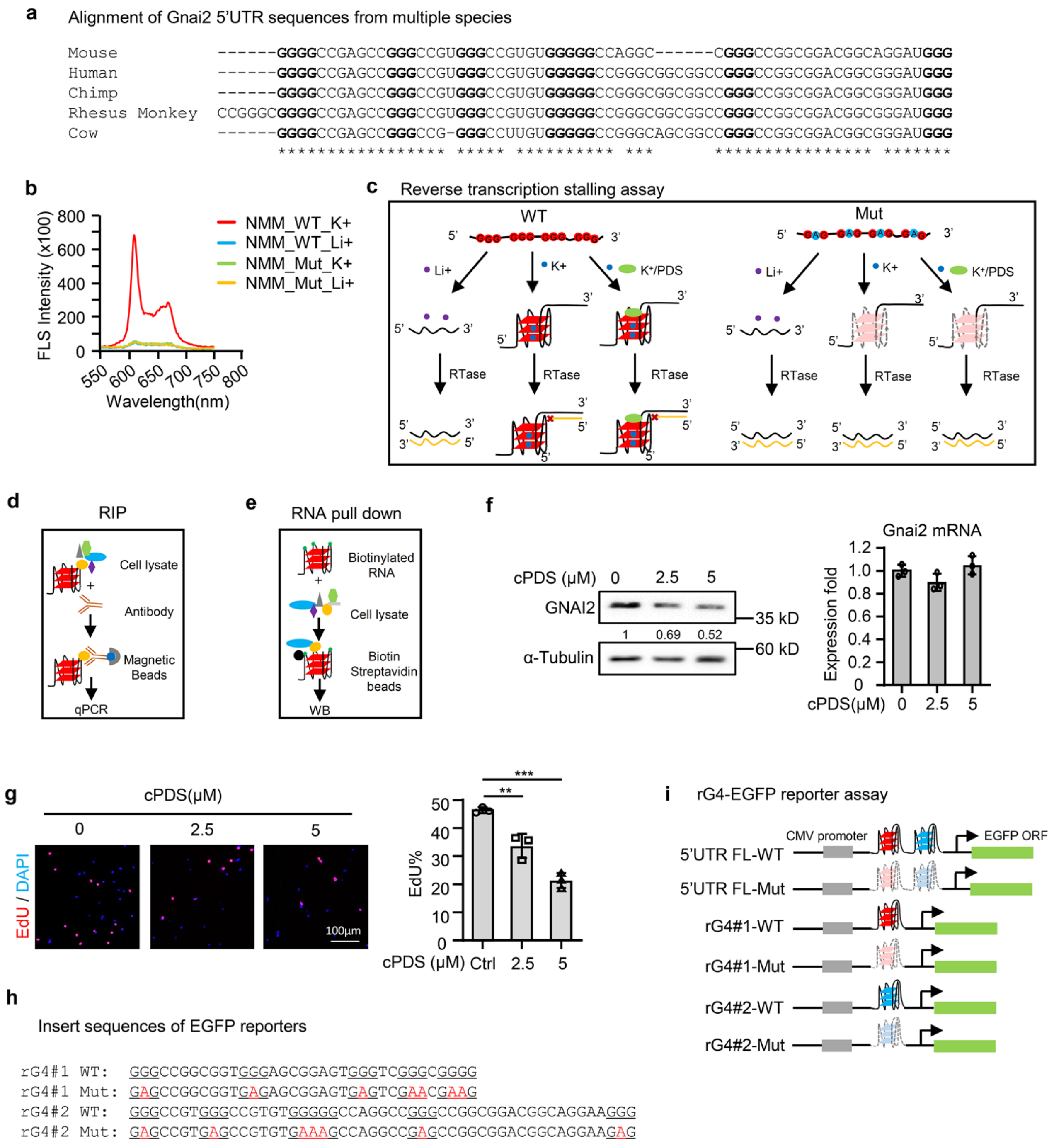
a
Alignment of Gnai2 5'UTR sequences from multiple species
Mouse ------GGGGCCGAGCCGGGCCGUGGGCCGUGUGGGGGCCAGGC------CGGGCCGGCGGACGGCAGGAUGGG
Human ------GGGGCCGAGCCGGGCCGUGGGCCGUGUGGGGGCCGGGCGGCGGCCGGGCCGGCGGACGGCGGGAUGGG
Chimp ------GGGGCCGAGCCGGGCCGUGGGCCGUGUGGGGGCCGGGCGGCGGCCGGGCCGGCGGACGGCGGGAUGGG
Rhesus Monkey CCGGGCGGGGCCGAGCCGGGCCGUGGGCCGUGUGGGGGCCGGGCGGCGGCCGGGCCGGCGGACGGCGGGAUGGG
Cow ------GGGGCCGAGCCGGGCCG-GGGCCUUGUGGGGGCCGGGCAGCGGCCGGGCCGGCGGACGGCGGGAUGGG
b
FLS Intensity (x100)
Wavelength(nm)
NMM_WT_K+
NMM_WT_Li+
NMM_Mut_K+
NMM_Mut_Li+
c
Reverse transcription stalling assay
WT
Mut
Li+
K+
K+/PDS
RTase
d
RIP
Cell lysate
Antibody
Magnetic Beads
qPCR
e
RNA pull down
Biotinylated RNA
Cell lysate
Biotin Streptavidin beads
WB
f
cPDS (μM) 0 2.5 5
GNAI2
1 0.69 0.52
α-Tubulin
35 kD
60 kD
Gnai2 mRNA
Expression fold
cPDS(μM) 0 2.5 5
g
cPDS(μM)
0 2.5 5
EdU / DAPI
100μm
EdU%
cPDS (μM) Ctrl 2.5 5
**
***
h
Insert sequences of EGFP reporters
rG4#1 WT: GGGCCGGCGGTGGGAGCGGAGTGGGTCGGGCGGGG
rG4#1 Mut: GAGCCGGCGGTGAGAGCGGAGTGAGTCGAACGAAG
rG4#2 WT: GGGCCGTGGGCCGTGTGGGGGCCAGGCCGGGCCGGCGGACGGCAGGAAGGG
rG4#2 Mut: GAGCCGTGAGCCGTGTGAAAGCCAGGCCGAGCCGGCGGACGGCAGGAAGAG
i
rG4-EGFP reporter assay
CMV promoter
EGFP ORF
5'UTR FL-WT
5'UTR FL-Mut
rG4#1-WT
rG4#1-Mut
rG4#2-WT
rG4#2-Mut

Supplementary Figure 8. DHX36 regulates *Gnai2* mRNA translation by unwinding the rG4 formed at the 5'UTR. a The alignment of *Gnai2* 5'UTR sequences from multiple species shows the conserved G rich regions. **b** WT and Mut *Gnai2* 5'UTR RNA stained with G4 ligand NMM under the treatment of 150 mM Li+ or K+ and excited at a wavelength of 394 nm. FLS was collected from 550 nm to 750 nm. Red line: WT RNA treated with K+; blue line: WT RNA treated with Li+; green line: Mut RNA treated with K+; orange line: Mut RNA treated with Li+. **c** Schematic illustration of Reverse transcriptase stalling (RTS) assay. WT and Mut *Gnai2* 5'UTR RNAs were treated with 150 mM Li+, 150 mM K+ or 150 mM K+ plus 2 μM PDS. Reverse transcriptase was stalled at the rG4 formation site to form reverse stalling sites (RTS). **d** Illustration of RNA immunoprecipitation (RIP) and **e** RNA pull-down assay. **f** Gnai2 protein and mRNA level were detected by Western blot and qRT-PCR in C2C12 myoblast cells treated with 2.5 or 5 μM cPDS for 24 h. Data represent the average of three independent experiments ± s.d. **g** SCs were treated with 2.5 or 5 μM cPDS for 24 h. EdU was then added to the culture medium 4 h before staining. The percentage of EdU incorporation was quantified from 10 randomly selected fields/sample. Scale bar=100 μm. Data represent the average of three independent experiments ± s.d. Student's t-test (two-tailed unpaired) was used to calculate the statistical significance: **, $P < 0.01$, P=0.0093; ***, $P < 0.001$, P=0.000048. **h** Sequences of WT and mutated rG4#1 and rG4#2 used for inserting into the EGFP reporter in Fig. 7. **i** Cloning scheme of the plasmids used in the reporter assay. WT or Mut sequences of full length 5'UTR, rG4#1, or rG4#2 only were cloned upstream of EGFP open reading frame in pEGFP-N1 vector. All GGGs were mutated to GAG in the Mut sequences. Source data are provided as a Source Data file.

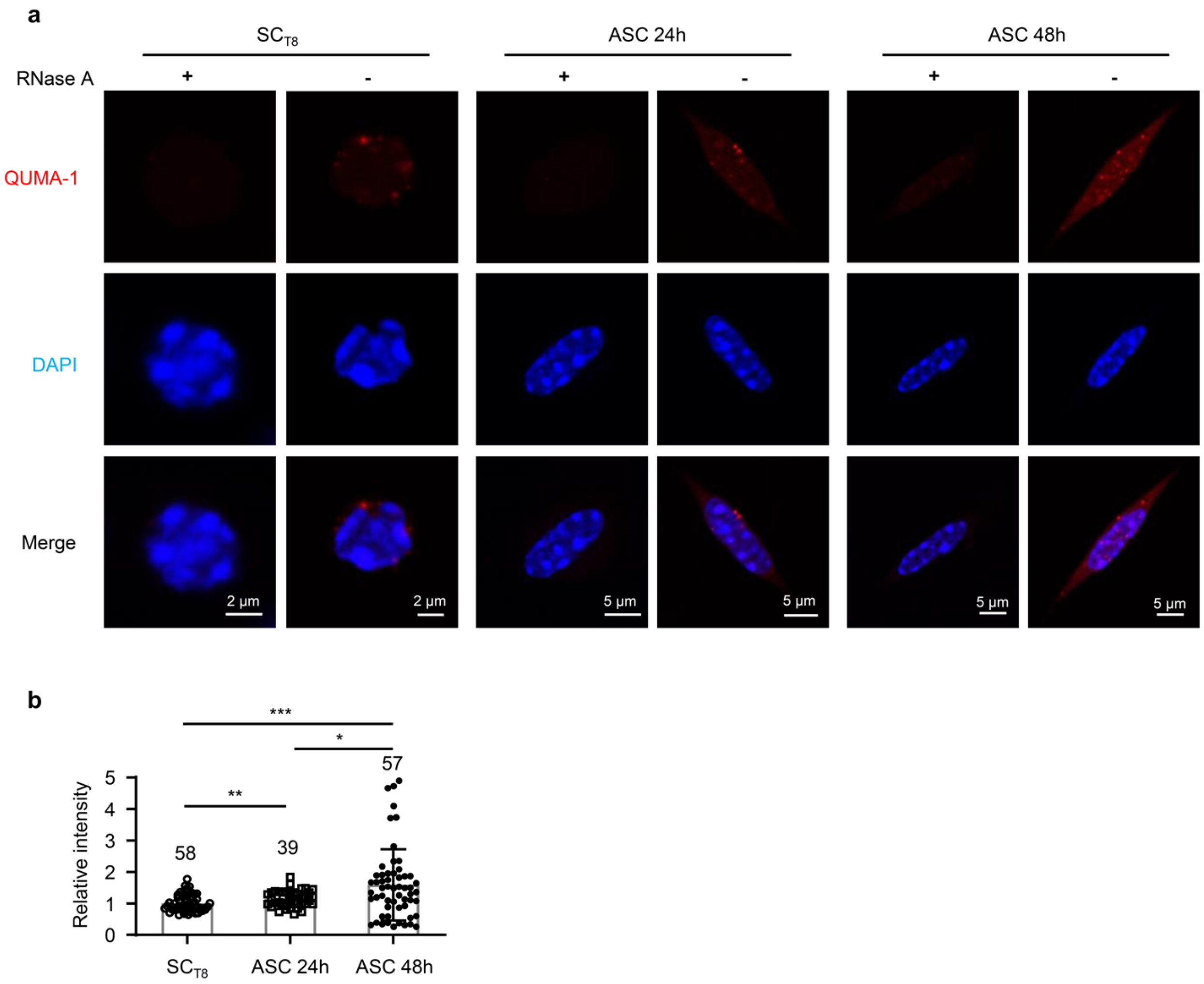

Supplementary Figure 9. rG4 staining in SCs. a SC_{T8}, ASC 24h and ASC 48h were stained with an rG4 specific fluorescent ligand QUMA-1. Cells treated with RNase A was used as negative controls. **b** Normalized rG4 fluorescence signals which were calculated from indicated number of cells by Matlab R2014b using in-house scripts. The average intensity from SC_{T8} cells was set as 1. Data represent the average of indicated cell No. from three independent experiments ± s.d. Student's t-test (two-tailed unpaired) was used to calculate the statistical significance: * $P < 0.05$, P=0.017, ** $P < 0.05$, P=0.0088, *** $P < 0.001$, P=0.000174. Source data are provided as a Source Data file.

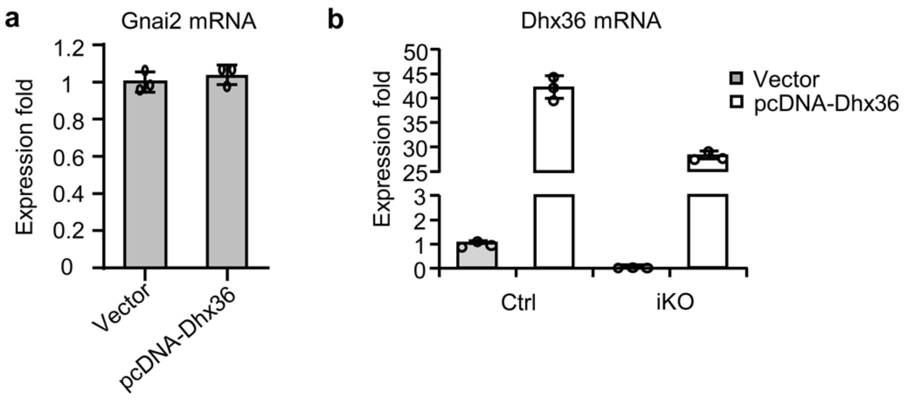

Supplementary Figure 10. DHX36 overexpression in SCs promoted cell proliferation. a *Gnai2* mRNA level was detected by qRT-PCR in Dhx36 KO myoblast cells transfected with a negative control vector (Vector) or pcDNA-Dhx36 overexpressing plasmid (pcDNA-Dhx36). *18s rRNA* was used as normalization control. Data represent the average of three independent experiments ± s.d. **b** The overexpression of DHX36 was confirmed by qRT-PCR in Ctrl and Dhx36 iKO SCs transfected with negative control vector (Vector in gray bars) or pcDNA-Dhx36 overexpressing plasmid (pcDNA-Dhx36 in white bars). *18s rRNA* was used as normalization control. Data represent the average of three independent experiments ± s.d. Source data are provided as a Source Data file.
