# Supplementary material for: Translational control by DHX36 binding to 5′UTR G-quadruplex is essential for muscle stem-cell regenerative functions
Source: Nat Commun. 2021 Aug 19;12:5043. doi: 10.1038/s41467-021-25170-w (PMC8377060; doi:10.1038/s41467-021-25170-w)
Supplement: Supplementary file 3 — Reporting Summary [file 41467_2021_25170_MOESM3_ESM.pdf]

nature research

Corresponding author(s): Huating Wang

Last updated by author(s): May 26, 2021

Reporting Summary

Nature Research wishes to improve the reproducibility of the work that we publish. This form provides structure for consistency and transparency in reporting. For further information on Nature Research policies, see our Editorial Policies and the Editorial Policy Checklist.

Statistics

For all statistical analyses, confirm that the following items are present in the figure legend, table legend, main text, or Methods section.

n/a	Confirmed	
☐	☒	The exact sample size (*n*) for each experimental group/condition, given as a discrete number and unit of measurement
☐	☒	A statement on whether measurements were taken from distinct samples or whether the same sample was measured repeatedly
☐	☒	The statistical test(s) used AND whether they are one- or two-sided<br>*Only common tests should be described solely by name; describe more complex techniques in the Methods section.*
☒	☐	A description of all covariates tested
☒	☐	A description of any assumptions or corrections, such as tests of normality and adjustment for multiple comparisons
☐	☒	A full description of the statistical parameters including central tendency (e.g. means) or other basic estimates (e.g. regression coefficient) AND variation (e.g. standard deviation) or associated estimates of uncertainty (e.g. confidence intervals)
☐	☒	For null hypothesis testing, the test statistic (e.g. *F*, *t*, *r*) with confidence intervals, effect sizes, degrees of freedom and *P* value noted<br>*Give P values as exact values whenever suitable.*
☒	☐	For Bayesian analysis, information on the choice of priors and Markov chain Monte Carlo settings
☒	☐	For hierarchical and complex designs, identification of the appropriate level for tests and full reporting of outcomes
☐	☒	Estimates of effect sizes (e.g. Cohen's *d*, Pearson's *r*), indicating how they were calculated

Our web collection on statistics for biologists contains articles on many of the points above.

Software and code

Policy information about availability of computer code

Data collection	qRT-PCR were conducted using the Light Cycler ®480 Real-Time PCR System (Roche Applied Science);<br>Microscopy pictures were acquired with Leica microscope system DM 6000B and laser scanning confocal microscope (Carl ZEISS LSM 880). Leica LAS AF software (LAS AF2.6.3) was used for the acquisition of images from Leica microscope. ZEN 3.2 (blue edition) software was used to acquisition of images from confocal microscope;<br>BD FACSVerse flow cytometer, BD FACSAria Fusion Cell Sorter and BD FACSDiva (version 8.0.1, BD Biosciences) were used for the acquisition of flow cytometry data;<br>Fluorescence spectroscopy data was collected by HORIBA FluoroMax-4;<br>CD spectroscopy data was collected using a Jasco CD J-150 spectrometer.
Data analysis	GraphPad Prism version 8.2 was used to data analysis.<br>Leica LAS AF software (LAS AF2.6.3) was used for the analysis of images from Leica microscope.<br>ImageJ 1.50i (National Institutes of Health) were used to Western blot band intensities quantification.<br>WinMDI 2.8. was used to analyze flow cytometry data.<br>Spectra Manager Suite (Jasco Software) was used to analyze the CD spectroscopy data.<br>The analysis associated with CLIP-seq/Polysome profiling were conducted mainly using custom code. Basic NGS-processing softwares including TopHat2 (v2.1.1), Cufflinks (v2.2.1), Bowtie2 (v2.3.3.1) were used. Additionally, for CLIP-seq motif analysis, FIMO (v4.11.4) /MEME (v4.11.4) / RNAshapes (v2.1.6) were used for the inference of DNA motifs and snoSCAN (Server v1.0) for potential snoRNA prioritaztion. The rG4 forming motifs/AREs were identified via custom code (deposited on https://github.com/jieyuanCUHK/DHX36_paper). RNAfold (v2.4.10) was used for Minimum Free Energies calculation based on rG4 forming motifs.

For manuscripts utilizing custom algorithms or software that are central to the research but not yet described in published literature, software must be made available to editors and reviewers. We strongly encourage code deposition in a community repository (e.g. GitHub). See the Nature Research guidelines for submitting code & software for further information.

Data

Policy information about availability of data

All manuscripts must include a data availability statement. This statement should provide the following information, where applicable:

- Accession codes, unique identifiers, or web links for publicly available datasets
- A list of figures that have associated raw data
- A description of any restrictions on data availability

RNA-seq data of quiescent satellite cells (SCT0) isolated after in situ fixation, freshly isolated SCs without prior fixation (SCT8) (fixed after isolation) and SCs cultured for 24 h, 48 h or 72 h is deposited in Gene Expression Omnibus (GEO) database with the accession codes GSE175501. CLIP-seq and Polysome profiling data used in this study have been deposited in Gene Expression Omnibus (GEO) database under the accession codes GSE151124.
Fig. 1b, 1c, 1d, 1h, 1i, 1j, 1l, 1m, 2c, 2f, 2g, 3a-j, 7c, 7d, 7f, 7g, 8a-i, 9a-h, Supplementary Fig. 1b-g, 2a-g, 4d-g, 8b, 8f, 8g, 9b, 10a-b have associated raw data. All other data supporting the findings of this study are available from the corresponding author on reasonable request.

Field-specific reporting

Please select the one below that is the best fit for your research. If you are not sure, read the appropriate sections before making your selection.

[x] Life sciences   [ ] Behavioural & social sciences   [ ] Ecological, evolutionary & environmental sciences

For a reference copy of the document with all sections, see nature.com/documents/nr-reporting-summary-flat.pdf

Life sciences study design

All studies must disclose on these points even when the disclosure is negative.

Sample size	At least three biological replicates per group (detailed n is indicated in the figure or figure legends) were collected to perform statistical testing. No statistical test was used to determine sample size. For CLIP-seq and polysome profiling, two biological replicates per group were used. For RNA-seq of muscle satellite cells, one sample per group was used.
Data exclusions	No data or samples were excluded from the analysis
Replication	All experimental data was repeated in multiple biological independent experiments as described in the legend, method and source data except the RNA-seq of SCs, for which data of one experiment was shown.
Randomization	For all the animal experiments, we used the the same age and sex of Control and KO mice from the same litter whenever possible. For cell experiments, we randomly counted multiple fields or cells per group for calculation.
Blinding	For immunofluorescence data collection , we performed the experiments in a blinded way. We randomly counted multiple fields per group and calculated the number of positively stained cells per field. For immunofluorescence intensity quantification, we were blinded to cell allocation during data analysis. For RNA analyses using qRT-PCR and Protein analyses using Western blotting were not performed blind to load samples by order.

Reporting for specific materials, systems and methods

We require information from authors about some types of materials, experimental systems and methods used in many studies. Here, indicate whether each material, system or method listed is relevant to your study. If you are not sure if a list item applies to your research, read the appropriate section before selecting a response.

Materials & experimental systems

n/a	Involved in the study	
[ ]	[x]	Antibodies
[ ]	[x]	Eukaryotic cell lines
[x]	[ ]	Palaeontology and archaeology
[ ]	[x]	Animals and other organisms
[x]	[ ]	Human research participants
[x]	[ ]	Clinical data
[x]	[ ]	Dual use research of concern

Methods

n/a	Involved in the study	
[x]	[ ]	ChIP-seq
[ ]	[x]	Flow cytometry
[x]	[ ]	MRI-based neuroimaging

Antibodies

Antibodies used	Antibodies of DHX36 (Abcam ab70269; 1:5,000), α-Tubulin (Santa Cruz Biotechnology sc-23948; 1:5000), MyoD (Santa Cruz Biotechnology sc-760; 1:1000), Gnai2 (Abcam ab157204; 1:5,000) , CCND1 (Santa Cruz Biotechnology sc-718; 1:5000), CCNA1 (Santa Cruz Biotechnology sc-596; 1:5000), p-S6 (Cell signaling technology #4858; 1:5,000), HnrnpL (Santa Cruz Biotechnology sc-28726;

1:5,000), Dhx9 (Santa Cruz Biotechnology sc-137232; 1:5,000) were used for Western blot. For immunofluorescence staining of cultured cells and myofibers, antibodies of DHX36 (Proteintech 13159-1-AP), MyoD (Santa Cruz Biotechnology sc-760; 1:200) and Myogenin (Santa Cruz Biotechnology sc-576; 1:200); Pax7 (Developmental Studies Hybridoma Bank; 1:50) and MF20 (Developmental Studies Hybridoma Bank; 1:50); eMyHC (Sigma-AldrichLeica NCL-MHC-d; 1:200) and laminin (Sigma-Aldrich L9393; 1:800); Ki67 (Santa Cruz Biotechnology sc-15402; 1:200); MyoD (Dako M3512; 1:200)for staining of muscle cryosections or SCs.

Validation

All antibodies used are commercially available and the applications have been tested by the manufacturers with the validation information for application and antigen specificity being provided in the respective data sheets from the manufacturers or on the manufacturers' websites.

Eukaryotic cell lines

Policy information about cell lines

Cell line source(s)	Mouse C2C12 myoblast cells (CRL-1772) and 293T (CRL-3216) cells were obtained from American Type Culture Collection (ATCC).
Authentication	Cell lines were procured from commercial source and therefore, not authenticated.
Mycoplasma contamination	All cell lines were tested as negative for mycoplasma contamination.
Commonly misidentified lines (See ICLAC register)	No commonly misidentified lines were used in this study.

Animals and other organisms

Policy information about studies involving animals; ARRIVE guidelines recommended for reporting animal research

Laboratory animals	Pax7CreER (Pax7tm1(cre/ERT2)Gaka) and Tg: Pax7-nGFP mouse strains were kindly provided by Dr. Shahragim Tajbakhsh. Pax7Cre (Pax7tm1(cre)Mrc) mouse was kindly provided by Dr. Charles Keller. ROSA-EYFP mouse was provided by Jackson Laboratory. Dhx36fl/fl strain was kindly provided by Dr. Zhongzhou Yang with the authorization of Dr. Yoshikuni Nagamine who originally generated this mouse strain. Pax7Cre and Dhx36fl/fl mice were mated to generate Dhx36 conditional KO (Dhx36 cKO) mice (Ctrl: Pax7Cre/+; Dhx36+/+, cKO: Pax7Cre/+; Dhx36fl/fl). Pax7CreER mouse was crossed with ROSAEYFP mice to generate the Pax7CreER; ROSAEYFP reporter mice. The Dhx36 inducible conditional KO (Dhx36 iKO) mice with EYFP reporter (Ctrl: Pax7CreER/+; ROSAEYFP/+; Dhx36+/+, iKO: Pax7CreER/+; ROSAEYFP/+; Dhx36fl/fl) were generated by crossing Pax7CreER; ROSAEYFP with Dhx36fl/fl mice. 8-12 weeks old mice were used for experiments. Either male or female mice were used with the comparable pairs of the same gender.
Wild animals	No wild animals were used in this study.
Field-collected samples	No field-collected samples were used in this study.
Ethics oversight	All iKO mice handling procedures and protocols were approved by the Animal Ethics Committee at Chinese University of Hong Kong. The animal procedures involved cKO mice were supervised by the Ethical Committee of Animal Experimentation of the PRBB (CEEA-PRBB) and previously authorized by the corresponding Catalan Government committee, the Section for the Domestic Animal Protection, General Direction of Environmental and Nature Politics, Department of Territory and Sustainability.

Note that full information on the approval of the study protocol must also be provided in the manuscript.

Flow Cytometry

Plots

Confirm that:

- [x] The axis labels state the marker and fluorochrome used (e.g. CD4-FITC).
- [x] The axis scales are clearly visible. Include numbers along axes only for bottom left plot of group (a 'group' is an analysis of identical markers).
- [x] All plots are contour plots with outliers or pseudocolor plots.
- [x] A numerical value for number of cells or percentage (with statistics) is provided.

Methodology

Sample preparation	Muscle satellite cells preparation is described in the "Methods" section.<br>PI/FACS (cell cycle) analysis:<br>Harvest cells and wash with PBS once. Suspend cells in 500ul of PBS containing 0.1% Glucose and immediately add 5 ml of cold 70% ETOH. Mix immediately and keep at 4 °C for overnight. Spin cells down and wash with PBS. Suspend in 300ul propidium iodide solution and add 20ul of 10mg/ml RNase. Mix and incubate at 37°C for 45min. Transfer to FACS tubes and run samples on flow cytometer and approximately 10,000 events were analysed for each sample.
Instrument	BD FACSAria Fusion Cell Sorter and BD FASC Calibur

Software	WinMDI 2.8. and BD FACSDiva
Cell population abundance	Muscle satellite cells sorting:<br>We stained isolated cells by FACS with Pax7 antibody which is the marker of satellite cells and found 98% cells were Pax7 positive.
Gating strategy	Muscle satellite cells sorting:<br>Gating strategy was done according to previously reported protocol which sorted out the GFP/YFP positive population.<br>PI/FACS (cell cycle) analysis:<br>Set appropriate FSC vs SSC gates to exclude debris and cell aggregates. Collect Propidium iodide fluorescence in FL2.

☒ Tick this box to confirm that a figure exemplifying the gating strategy is provided in the Supplementary Information.
