# Supplementary material for: Translational control by DHX36 binding to 5′UTR G-quadruplex is essential for muscle stem-cell regenerative functions
Source: Nat Commun. 2021 Aug 19;12:5043. doi: 10.1038/s41467-021-25170-w (PMC8377060; doi:10.1038/s41467-021-25170-w)
Supplement: Supplementary file 4 — Description of Additional Supplementary Files [file 41467_2021_25170_MOESM4_ESM.pdf]

Description of Additional Supplementary Files

File name: Supplementary Data 1

Description: CLIP-seq analysis of DHX36 RNA binding in C2C12 myoblasts.

File name: Supplementary Data 2

Description: Translational profiling analysis in WT or Dhx36 KO C2C12 myoblasts.

File name: Supplementary Data 3

Description: mRNA abundance analysis in WT or Dhx36 KO C2C12 myoblasts.

File name: Supplementary Data 4

Description: Information of oligonucleotides and primers used in the study.
